# Body composition impacts appetite regulation in middle childhood. A prospective study of Norwegian community children

**DOI:** 10.1186/s12966-017-0528-5

**Published:** 2017-05-30

**Authors:** Silje Steinsbekk, Clare H. Llewellyn, Alison Fildes, Lars Wichstrøm

**Affiliations:** 1 Department of Psychology, Norwegian University of Science and Technology (NTNU), Dragvoll, 7491 Trondheim, Norway; 2 Department of Behavioural Science & Health, University College London, 1-19 Torrington Place, London, WC1E 7HB UK; 3 School of Psychology, University of Leeds, Leeds, LS2 9JT UK; 4 NTNU Social Research, 7491 Trondheim, Norway

**Keywords:** Body composition, Fat mass, Fat-free mass, Appetite, Food responsiveness, Satiety responsiveness, Eating behavior, Longitudinal design

## Abstract

**Background:**

Research suggests a role for both fat mass and muscle mass in appetite regulation, but the longitudinal relationships between them have not yet been examined in children. The present study therefore aimed to explore the prospective relationships between fat mass, muscle mass and the appetitive traits food responsiveness and satiety responsiveness in middle childhood.

**Methods:**

Food responsiveness and satiety responsiveness were measured using the parent-reported Children’s Eating Behavior Questionnaire in a representative sample of Norwegian 6 year olds, followed up at 8 and 10 years of age (*n* = 807). Body composition was measured by bioelectrical impedance.

**Results:**

Applying a structural equation modeling framework we found that higher fat mass predicted greater increases in food responsiveness over time, whereas greater muscle mass predicted decreases in satiety responsiveness. This pattern was consistent both from ages 6 to 8 and from ages 8 to 10 years.

**Conclusions:**

Our study is the first to reveal that fat mass and muscle mass predict distinct changes in different appetitive traits over time. Replication of findings in non-European populations are needed, as are studies of children in other age groups. Future studies should also aim to reveal the underlying mechanisms.

## Background

Two distinct aspects of appetite that are neurologically dissociable have been studied in detail in relation to weight and weight gain: food responsiveness (FR) and satiety responsiveness (SR) [1, 2]. FR is the tendency to want to eat (or eat more) in response to the sight or smell of highly palatable food (i.e. eating for pleasure) [3–6], and is governed by the hedonic appetite system [7]. Satiety responsiveness (SR) characterizes ‘fullness’ sensitivity (i.e. how quickly an individual fills up once they start eating, and how long they remain full for before they want to start eating again) [3, 4], and is thought to reflect the homeostatic appetite system that regulates hunger and satiety according to energy needs, primarily via the melanocortin pathway [8, 9]. Although homeostatic and hedonic mechanisms are rooted in neurologically dissociable systems, there is considerable interplay between the two. The homeostatic system can modulate the hedonic control of appetite and vice versa. For example, leptin and insulin can dampen hedonic responses to food though action on dopaminergic neurons in the mesolimbic pathway [10], while grehlin facilitates reward processing [11]. At the same time reward processes are activated in response to the consumption of palatable food and can override homeostatic satiety mechanisms in the hypothalamus to prolong eating [12].

The Child Eating Behavior Questionnaire (CEBQ) is a widely used parent-report psychometric measure of appetitive characteristics in children, including FR and SR, which has been validated using laboratory measures of eating [13]. FR and SR show moderate tracking from early to late childhood [14, 15], indicating that they are relatively stable traits, although self-regulation of eating (i.e. low FR, high SR) decreases as children get older [14, 16]. A wealth of research with the CEBQ has established that FR is positively, whereas SR is negatively associated with energy intake, weight, and weight gain [3–5, 17–19]. The majority of research in this area has been cross-sectional [3–5, 17], but a few prospective studies have provided evidence that greater FR and lower SR predict greater weight gain [15, 20, 21]. However, research with the CEBQ to date has generally aimed to identify predictors of overweight; while the opposite direction of influence, i.e. from weight to appetite, has received little attention. Notably though, adipose tissue itself plays an important role in homeostatic regulation of appetite, with leptin (the ‘satiety hormone’) being the major signal that informs the brain about the state of the body’s energy status [22, 23]; as adipose tissue increases, leptin does too, thus downregulating hunger.

Interestingly as well, studies have implicated fat free mass in appetite regulation. A study examining the effect of exercise on appetite regulation in overweight adults found that fat-free mass, but not fat mass, was related to meal size and greater daily energy intake; an association that was conserved over time [24]. In addition, a more recent study of obese adolescents found skeletal muscle mass to be the strongest predictor of energy intake [25]. The association between energy intake and fat free mass has also been found in a community sample of adolescent boys [26]. It has been hypothesized that the increased energy needed to maintain lean tissue (and in particular, muscle mass) is signaled physiologically by fat-free mass, which upregulates appetite [22]. In support of this, resting metabolic rate (RMR), largely determined by fat-free mass, has been associated with increased appetite, characterized by increased meal size and daily energy intake [27]. These and earlier findings [28–30] suggest a role for both fat mass and muscle mass in appetite regulation, which is reasonable given that muscle and adipose tissue are anatomically, biologically and pathologically related to each other [31]. In keeping with this, both fat-free mass and fat-mass were positively associated with laboratory measured energy intake in children [32].

However, the prospective relationships between fat mass, fat free mass and appetite have not yet been examined. Understanding the relationship between changes in body composition and the impact on appetite has important implications for weight management in both health and disease [22]. In addition, all previous studies on the effect of fat mass and fat-free mass on appetite regulation have been laboratory based. Although laboratory-based measures of appetite provide unparalleled detail, only a single ‘snapshot’ of eating behavior is captured, and behavior is subject to any extraneous factors at play during the test meal. While standardized psychometric measures of appetite (such as the CEBQ) lose the objectivity of laboratory-based observations, they have the advantage of characterizing habitual eating behavior (i.e. measuring the ‘trait’ rather than a ‘state’), which is likely responsible for individual differences in weight. Examination of the relationships between variation in body-composition and appetitive traits captured psychometrically, would add importantly to the research base.

The present study therefore aimed to examine the prospective relationships between fat mass, muscle mass and the appetitive traits FR and SR measured using the CEBQ in a representative sample of Norwegian 6 year olds, followed up at 8 and 10 years of age. We recently showed that children with greater body mass index (BMI) at age 6 displayed higher FR and lower SR 2 years later, when initial levels of FR and SR were accounted for [15], but the contributions of fat mass and fat free mass were not examined. In the current study we hypothesize that greater fat mass will downregulate FR and upregulate SR over time, whereas increased fat free mass will upregulate FR and downregulate SR over time.

## Methods

### Participants and procedure

**Fig. 1:**
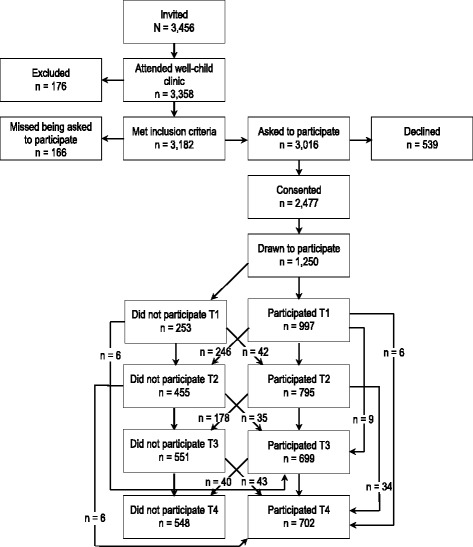
Sample recruitment and follow-up. Note. Number of participants at the different assessment points is based on the number of participants drawn to participate (*n* = 1250), minus those who did not participate at the respective measurement point (i.e., T1, T2, T3, T4)

The Trondheim Early Secure Study (TESS) comprises members of the 2003 and 2004 birth cohorts in Trondheim, Norway (*N* = 3456). A letter of invitation together with the Strengths and Difficulties Questionnaire (SDQ) 4–16 version [33], a screening assessment for emotional and behavioral problems, was sent to all children in the two birth cohorts’ homes. The SDQ was used because the primary aim of TESS was to assess mental health. Parents brought the completed SDQ when they attended the well-child clinic for the routine health check at age 4 years. As shown in Fig. 1, almost all children in the two cohorts appeared at the check-up (97.2%), thus the sample is effectively a community sample. Parents were informed about the present study by the health nurse, using procedures approved by the Regional Committee for Medical and Health Research Ethics Mid-Norway. As part of the primary TESS focus, children were allocated to four strata according to their SDQ scores (cut-offs: 0–4, 5–8, 9–11, and 12–40), and the probability of selection increased with increasing SDQ scores (0.37, 0.48, 0.70, and 0.89 in the four strata, respectively). At Time 1 (T1) the participants’ mean age was 4.7 years (SD = .30), at follow-ups two (T2) and four (T3) years later, children were aged 6.7 years (SD = 0.17) and 8.8 years (SD = 0.24), respectively. Because body composition was measured from T2 onwards, the present inquiry uses data collected at T2, T3, and T4 exclusively. Characteristics of the TESS participants are presented in Table 1. The sample, weighted to adjust for the oversampling just described, is comparable to the Norwegian parent population for the parents’ level of education [34] and children’s BMI [35].

**Table 1:** Sample characteristics at baseline

Characteristics	%
Gender of the child	
Male	49.8
Female	50.2
Weight status	
Overweight	9.3
Obese	0.4
Gender of the parent informant	
Male	18.9
Female	81.1
Ethnic origin of mother	
Norway	93.0
Western country	6.8
Other country	0.3
Ethnic origin of father	
Norway	93.0
Western country	6.5
Other country	0.5

Note: *BMI SDS* Body Mass Index Standard Deviation Score. Percentage of children with overweight/obesity are estimated based on the IOTF cut offs

### Measures

***Fat-mass and muscle mass*** was successfully measured in 595 participants at T2, 564 participants at T3 and 690 participants at T4 by bioelectrical impedance using a body composition analyzer (Tanita BC420MA). No specific instructions were given prior to the body composition measurements (e.g., intake, hydration). Participants were measured barefooted wearing light indoor clothing (Correction for light indoor clothing (0.5 kg) was applied). Muscle mass (i.e. bone-free lean tissue mass) rather than fat free mass was used because a newly published study showed skeletal muscle mass to be the strongest predictor of energy intake in adolescents [25].

***Food responsiveness (FR) and satiety responsiveness*** **(SR)** were measured by the Norwegian version of the parent completed Children’s Eating Behavior Questionnaire (CEBQ) [36]. Both these subscales consist of five items (FR: e.g. “If allowed to, my child would eat too much”, T2: α = .69; T3: α = .69; T4: α = .71) (SR: e.g., “My child gets full easily”, T2: α = .70; T3: α = .74; T4: α = .73) rated along a five point Likert scale (from ‘Never’ to ‘Always’).

### Statistical analyses

Data were analysed using structural equation modelling. An autoregressive cross lagged model was applied to test whether fat mass and muscle mass predicted FR and SR from ages 6 to 8, and 8 to 10 years. More specifically, FR and SR at ages 8 and 10 were regressed on fat mass and muscle mass at ages 6 and 8, respectively, accounting for initial levels of FR and SR and allowing for within time associations. The autoregressive paths reflect the unique stability of each of the variables (e.g. FR), whereas the cross-lagged paths estimate the relation between the different variables over time (e.g. FR and fat mass). Because fat mass and muscle mass influence each other [37], they were regressed on each other over time, and because height could impact both fat-mass and muscle mass, we adjusted for height in the model. Goodness-of-fit of the measurement and structural models was evaluated according to the recommendations of Marsh et al. [38, 39]. Wald tests of parameter restraints were used to examine whether the detected paths differed by gender, testing one path at a time.

Analyses were performed in Mplus 7.0 [40] using a full information maximum likelihood estimator under the assumption that data were not missing completely at random. This implies that analyses are performed on all available data, provided that cases have at least some values for the dependent variables (i.e., FR and SR at ages 6, 8, and 10). The analysis sample is therefore *n* = 807.We used sampling weights to adjust for the stratified sampling (i.e. undersampled children – those with low SDQ scores – were weighted up, whereas oversampled children – those with high SDQ scores – were weighted down) to provide true population estimates. Analyses revealed that attrition at age 8 was higher among children with higher percentage of body fat at age 6 (B = .20, SE = .10, *p* = .04), but the effect was small (Nagelkerke proxy R2 = .012, Cox & Snell = .007). Attrition at age 10 was not predicted by body composition at age 6 or 8.

## Results

Means, standard deviations and correlation coefficients between all study variables are presented in Table 2. Means for FR and SR (Table 2) correspond to earlier studies [14, 17] and mean fat mass and muscle mass was comparable to reference data from the US [41] and the UK [42].

**Table 2:** Bivariate correlations between all study variables

		Body fat (kg)		Muscles (kg)			Food responsiveness (FR)			Satiety responsiveness (SR)		
	Mean (SD)	Age 8	Age 10	Age 6	Age 8	Age 10	Age 6	Age 8	Age 10	Age 6	Age 8	Age 10
Body fat (kg) Age 6	4.38 (1.27)	0.82	0.74	0.65	0.64	0.62	0.31	0.35	0.36	−0.32	−0.30	−0.34
Body fat (kg) Age 8	5.32 (2.05)		0.88	0.58	0.61	0.62	0.27	0.37	0.38	−0.23	−0.31	−0.34
Body fat (kg) Age 10	6.97 (3.76)			0.54	0.54	0.64	0.21	0.29	0.38	−0.18	−0.23	−0.28
Muscles (kg) Age 6	17.98 (2.00)				0.87	0.82	0.21	0.27	0.23	−0.36	−0.35	−0.36
Muscles (kg) Age 8	23.47 (2.77)					0.86	0.21	0.29	0.27	−0.31	−0.34	−0.36
Muscles (kg) Age 10	38.47 (3.68)						0.18	0.27	0.29	−0.25	−0.27	-0.35
FR Age 6	1.90 (0.47)							0.65	0.61	−0.19	−0.19	-0.19
FR Age 8	1.87 (0.48)								0.68	−0.15	−0.23	-0.20
FR Age 10	1.89 (0.52)									−0.15	−0.24	-0.23
SR Age 6	2.92 (0.50)										0.63	0.57
SR Age 8	2.80 (0.53)											0.67
SR Age 10	2.75 (0.56)											

Note. *FR* Food responsiveness, *SR* Satiety responsiveness. All correlations were significant at *p* ≤ .001

Table 3 displays the main results of the autoregressive cross lagged model. The model showed an acceptable fit to the data (CFI = 0.95; TLI = 0.90, RMSEA =0.066). As shown in Table 3, there were significant paths from fat mass at ages 6 and 8 to FR at ages 8 and 10, respectively, and from fat-free mass at ages 6 and 8 to SR at ages 8 and 10, respectively. Because initial levels of FR and SR were accounted for, as were height, the findings indicate that higher fat mass predicted increases in FR over time (i.e. eating is increasingly triggered by external cues), whereas higher fat free mass predicted decreases in SR over time (i.e. less sensitivity to internal signals of fullness). Wald test of parameter constraints revealed no significant gender differences with regard to the paths detected (Age 6 muscles to SR age 8: Wald = .45, df = 1, *p* = .50; Age 8 muscles to SR age 10: Wald = .00, df = 1, *p* = .10; Age 6 fat to FR age 8: Wald = .70, df = 1, *p* = .40; Age 8 fat to FR age 10: Wald = 1.89, df = 1, *p* = .17).

**Table 3:** Estimated paths of the main autoregressive cross lagged model (*N* = 807)

	Food responsiveness age 8		Satiety responsiveness age 8			Food responsiveness age 10		Satiety responsiveness age 10	
	B (95% CI)	β	B (95% CI)	β		B (95% CI)	β	B (95% CI)	β
Fat mass age 6	.05^**^ (.02, .09)	.13	−.01 (−.05, .03)	−.02	Fat mass age 8	.04^**^ (.01, .06)	.15	−.02 (−.04, .01)	−.06
Muscle mass age 6	.01 (−.02, .05)	.06	−.06^**^ (−.10, −.02)	−.23	Muscle mass age 8	.01 (−.02, .04)	.06	−.05^***^ (.-.08, −.02)	−.26

Note. Baseline levels of appetitive traits and children’s height were adjusted for and within time correlations between all study variables and between time relations between muscle mass and fat mass were estimated, but are not displayed in the figure. Asterisks indicate the level of significance (^*^ = *p* < 05; ^**^ = *p* ≤ .01; ^***^ = *p* ≤ .001)

## Discussion

The present study aimed to examine prospectively the relationship between variation in fat and muscle mass with change in the appetitive traits FR and SR in children. We hypothesized that higher fat mass would predict decreases in FR and increases in SR over time, due to higher levels of the ‘satiety hormone’ leptin. On the other hand, we hypothesized that higher muscle mass would predict increases in FR and decreases in SR over time, due to increased energy requirements. Findings did not fully support either hypothesis. Contrary to what we expected, higher fat mass predicted greater increases in FR, but did not significantly affect SR; whereas greater muscle mass predicted decreases in SR, but not increases in FR. Thus, although fat mass and muscle mass are closely linked biologically [31], they were found to be independently related to appetite. The above-noted pattern was consistent both from ages 6 to 8 and from ages 8 to 10 years. Our findings accord with a laboratory study of 4–6 year olds showing both fat mass and fat-free mass to be positively associated with energy intake [32]. Because laboratory measures of eating are proxies of eating in free-living conditions, our results extend earlier findings by showing fat mass and fat-free mass prospectively predict appetite *traits*, i.e. dispositions towards food shown to be relatively stable. Although we did not measure energy intake, the FR and SR scales of the CEBQ have been validated using laboratory measures of eating behavior [13], and both are related to energy intake [1]. As far as we can determine, this is the first study to show that fat mass and muscle mass predict distinct changes in *different* appetitive traits over time. Our findings might suggest that fat mass impacts the hedonic appetite control system, whereas muscle mass impacts the homeostatic appetite control system, although such assumption needs to be tested using more direct measures of hedonic and homeostatic control systems. Be aware that we applied a parent-reported measure of children’s appetite and thus did not capture direct measures of hunger or fullness.

The observation that increased fat-mass predicted increases in FR over time is at odds with what we know about the role of leptin having an inhibitory effect on food intake. However, this hypothesis is based on observations of lean individuals [22, 30], but leptin’s inhibitory effect weakens as fat mass increases (so-called ‘leptin resistance’), and in obese individuals leptin fails to suppress food intake [22]. The relationship between fat mass and appetite regulation is therefore complex; and it may differ for adults and children, who are still growing, and undergoing continuing changes in body composition due to growth. The notion that accumulating fat mass may fail to suppress food intake and permit more eating may apply to some children, possibly explaining why we found a positive association between fat mass and FR in children. In addition, as far as we are aware the relationship between fat mass and appetite has only been observed in the context of laboratory-based conditions. But energy intake during a test meal does not necessarily capture enduring appetitive traits that show variation over time, with children on the whole increasing in their food responsiveness as they mature.

We also observed that muscle mass specifically predicted a decline in children’s SR from the age of 6 to 8 as well as from age 8 to 10 years, adjusted for baseline levels of SR, but did not affect FR. This might suggest that the physiological signaling of muscle mass primarily impacts the homeostatic appetite control system, thereby altering levels of hunger and fullness, an assumption that warrants further testing. Nevertheless, this observation makes sense given the increased energy needs of children with higher muscle mass, and supports Blundell et al.’s proposition that fat-free mass affects energy intake primarily through control of meal size (a marker of satiation), shown in their studies of obese adults [24]. We add to this by showing the same to apply – prospectively – in a population sample of children. Increased muscle mass did not affect FR, however, which is more indicative of the hedonic appetite system. Future studies are needed to test whether muscle mass primarily impacts the homeostatic appetite control system, whereas fat mass impacts the hedonic system, although as noted by Blundell et al. [22], relationships between biological and behavioral variables must be established before potential biological explanations can be revealed. Future research should aim to reveal the underlying mechanisms, and whether different factors are at play in within-time vs long-term regulation of appetite. Studies should also be conducted to test whether the prospective associations revealed in our study can be replicated using objectively measured energy intake.

Some limitations must be addressed. Because the TESS sample is of European descent, replication of findings in non-European populations are needed, as are studies of children in other age-groups. Further, although the present sample is comparable to the Norwegian population as regards children’s BMI [35], replication in other populations with higher rates of overweight, as well as in overweight and obese samples is needed. Appetite traits were measured using a questionnaire, it is therefore a risk that social desirability may bias parent ratings. On the other hand, the advantage of using questionnaires rather than laboratory tests is the cost-effectiveness in the context of large samples and the potential to tap consistent behavioral style rather than behavior on a single occasion [43]. It should also be noted that the questionnaire used here has been validated against laboratory-reported measures of eating behavior [13]. Finally, using gold-standard measures of body composition such as dual-energy X-ray absorptiometry (DEXA) would have been preferable, but is too costly to be applied in such large samples as the present. Notably though, bioimpedance analyses of children’s body composition is found to be valid as tested against DEXA [44] and Tanita scales are considered accurate and efficient means of assessing body composition in epidemiological studies of elementary school children [44–46].

## Conclusions

Our study showed that muscle mass and fat mass prospectively predict changes in distinct aspects of appetite – muscle mass predicts decreases in SR and fat mass predicts increases in FR. Children with relatively higher levels of body fat seem to be increasingly triggered by external food cues over a 2 year span (from age 6 to 8 and from age 8 to 10), and children with a relatively larger muscle mass have blunted fullness in response to food over time, the latter possibly reflecting increasingly larger meal sizes. These two paths potentially represent different trajectories. The fat mass – FR path may reflect dysregulated hedonic appetite in response to accumulation of adiposity and an increased risk of overweight (a ‘vicious cycle’), whereas the muscle mass – SR path might reflect a normal and healthy adaptation in homeostatic appetite regulation that reflects increased energy needs (i.e. eating more because more energy is required with increasing levels of muscle mass), assumptions that need to be tested in future research.

## Abbreviations

BMI SDS: Body mass index standard deviation score
BMI: Body mass index
CEBQ: Children’s Eating Behavior Questionnaire
CFI: Comparative fit index
FR: Food responsiveness
RMSEA: Root mean square error of approximation
SR: Satiety responsiveness
TESS: The Trondheim Early Secure Study
TLI: Tucker-Lewis index
